# ALA-PpIX mediated photodynamic therapy of malignant gliomas augmented by hypothermia

**DOI:** 10.1371/journal.pone.0181654

**Published:** 2017-07-31

**Authors:** Carl J. Fisher, Carolyn Niu, Warren Foltz, Yonghong Chen, Elena Sidorova-Darmos, James H. Eubanks, Lothar Lilge

**Affiliations:** 1 Department of Medical Biophysics, University of Toronto, Toronto, ON, Canada; 2 Division of Molecular Imaging, Princess Margaret Cancer Centre, Toronto, ON, Canada; 3 Department of Radiation Oncology, University Health Network, Toronto, ON, Canada; 4 Department of Physiology, University of Toronto, Toronto, ON, Canada; 5 Division of Genetics and Development, Krembil Research Institute, Toronto Western Hospital, Toronto, ON, Canada; Massachusetts General Hospital, UNITED STATES

## Abstract

**Background:**

Malignant gliomas are highly invasive, difficult to treat, and account for 2% of cancer deaths worldwide. Glioblastoma Multiforme (GBM) comprises the most common and aggressive intracranial tumor. The study hypothesis is to investigate the modification of Photodynamic Therapy (PDT) efficacy by mild hypothermia leads to increased glioma cell kill while protecting normal neuronal structures.

**Methods:**

Photosensitizer accumulation and PDT efficacy *in vitro* were quantified in various glioma cell lines, primary rat neurons, and astrocytes. *In vivo* studies were carried out in healthy brain and RG2 glioma of naïve Fischer rats. Hypothermia was induced at 1 hour pre- to 2 hours post-PDT, with ALA-PpIX accumulation and PDT treatments effects on tumor and normal brain PDT quantified using optical spectroscopy, histology, immunohistochemistry, MRI, and survival studies, respectively.

**Findings:**

*In vitro* studies demonstrated significantly improved post-PDT survival in primary rat neuronal cells. Rat *in vivo* studies confirmed a neuroprotective effect to hypothermia following PpIX mediated PDT by T_2_ mapping at day 10, reflecting edema/inflammation volume reduction. Mild hypothermia increased PpIX fluorescence in tumors five-fold, and the median post-PDT rat survival time (8.5 days normothermia; 14 days hypothermia). Histology and immunohistochemistry show close to complete cellular protection in normal brain structures under hypothermia.

**Conclusions:**

The benefits of hypothermia on both normal neuronal tissue as well as increased PpIX fluorescence and RG2 induced rat survival strongly suggest a role for hypothermia in photonics-based surgical techniques, and that a hypothermic intervention could lead to considerable patient outcome improvements.

## Introduction

Glioblastoma Multiforme (GBM), comprising the most common and aggressive adult intracranial malignancy, has presented with a constant increasing incidence rate at over 2% per year between 1970 and 2000 [1]. While the median survival has increased during this time, it is still reported at just 14–16 months following standard therapies including surgical removal, radiation, and chemotherapy[2]. Various therapies including image guided surgical resection[3–5], gamma knife surgery[6], intensity modulated ionizing radiation therapy[7] and brachytherapy[8], or adjuvant chemotherapy[9] are being investigated for GBM. Even with the most aggressive treatment plans, the patients’ benefit has extended to a few months of additional survival time[10]. Encouraging is that the fraction of long-term survivors is increasing, possibly due to the realization that 98% tumor resection is required to afford long-term benefit to the patient[5].

Current therapeutic options are limited for non-surgically accessible GBM and those tumors which are proximal to eloquent areas of the brain, as most lengthy surgical interventions are not recommended. For these cases, photonics-based assistive tools including Photodynamic Therapy (PDT) and Fluorescence-Guided Resection (FGR) with the pro-drug Aminolevulinic Acid (ALA) are being investigated [11, 12]. One advantage of exogenous ALA-induced PpIX is that tumors show a preferential uptake of ALA, increasing synthesis of PpIX and retention thereof within the tumor mitochondria, versus normal intracranial tissues[13, 14]. This difference in PpIX concentration provides contrast between normal brain and tumor, enabling FGR and an increased therapeutic index for PDT.

Clinical studies demonstrated survival benefits for FGR or PDT adjuvant therapy of GBM. FGR adjuvant therapy is now well established in neuro-oncology [Stummer et al.], while PDT adjuvant therapy remains investigations outside of Japan using Talaporfin sodium as a photo-sensitizer [15]. For PDT survival time and progression-free survival have been extended by months to years compared to the current standard of care[11, 12, 16–18]. However, the pronounced improved outcomes were observed in small single-site clinical trials, with only limited improvements demonstrated in large multi-center trials[19]. One discrepancy could be heterogeneity in PpIX accumulation at the time of surgery, causing treatment failure and early recurrence [18].

Therefore, factors which improve PpIX accumulation can enhance FGR and PDT therapeutic efficacy in surgically inaccessible GBM. Our own *in vitro* study demonstrated that hypothermia (32–34°C) increased PpIX concentrations in glioma tumor cell lines, and improved FGR selectivity and the PDT therapeutic index [20]. Consistently, Dereski et al. demonstrated a thermal induced PDT-responsivity shift of healthy brain leading to a change in the therapeutic index[21], whereby hypothermia demonstrated neuroprotective effects for vascular-acting Photofrin mediated PDT[21]. Mild hypothermia has been shown to protect neurons following various *in vitro* and *in vivo* stroke-like insults[22], following hypoxia or glucose deprivation[23, 24] or acute neuronal injury[23, 24].

In this work, the PDT protective effect of hypothermia is demonstrated *in vitro* involving primary rat neuronal cells, rat and human glioma cell lines, and a human glioma stem cell line. *In vivo* the RG2 glioma model was utilized with whole-body hypothermia, assessing long-term survival, neuronal metrics, and quantitative MRI. The goal of the work was to increase the PDT selectivity in the brain adjacent to tumor, so that local micrometastasis can be effectively treated.

## Materials and methods

The University Health Network’s Animal Care Committee complying with regulations of the Canadian Council on Animal Care approved all procedures.

### Cell culture

Six (6) human glioblastoma cell lines (U373, U373vIII, U87, U87vIII, U343, and U118) and a differentiated rat glioma stem cell line (RG2) were grown in DMEM (Life Technologies, Carlsbad, CA, USA) supplemented with 10% Fetal Bovine Serum (Life Technologies, Carlsbad, CA, USA), 2mM glutamine (Life Technologies, Carlsbad, CA, USA), and Penicillin/Streptomycin (Life Technologies, Carlsbad, CA, USA). GS2 cells were cultured in McCoy’s5A (Life Technologies, Carlsbad, CA, USA) supplemented with 10% FBS, MEM Non-Essential Amino Acid Solution (Life Technologies, Carlsbad, CA, USA) and Penicillin/Streptomycin following the procedure by Gunther et al.[25].

#### Primary cortical cells and astrocytes isolation

Using a modified protocol presented by Brewer et al.[26], cortical cells were isolated from embryonic day 18 Wistar rats (Charles River Laboratories, Wilmington, MA, USA). The cell suspension was seeded in plating medium (Neurobasal medium containing 2% B-27 supplement, 1% fetal bovine serum, 0.5 mM L-glutamine, and 25 mM glutamic acid, Life Technologies, Carlsbad, CA, USA) at 30,000 cells/well in 96 well plates. After 96 hrs of isolation, cells were fed fresh growth medium (Neurobasal medium containing 2% B-27 supplement, 0.5mM L-Glutamine, (Life Technologies, Carlsbad, CA, USA) containing AraC (Cytosine arabinoside 4 μM, Sigma-Aldrich, Oakville, ON, CAN) and left to incubate for 48 hrs. Primary cortical neurons are selected by this procedure as verified by confocal microscopy on a subset of cultures, with versus without AraC, through staining of Map2. AraC was necessary to remove glial contaminates in the final culture. These cultures were maintained with new medium every 3–4 days, and used on days 12–14 after plating.

For the generation of primary astrocytes, day 1–2 postnatal pups were euthanized, their cortex separated and placed in ice-cold HBSS, to remove the meninges. After rinsing in HBSS, tissue was chopped into 1 mm^3^ cubes while being in a minimal quantity of media. Brain cubes were incubated with 0.05% Trypsin for 30 min at 37°C, dissociated with trituration 12–15 times using a polished Pasteur pipette, centrifuged at 1000 rpm for 5 minutes, re-suspended and triturated again followed by centrifugation. 350 μL phosphate-buffered saline with 100 μL Trypan Blue (Sigma–Aldrich) were mixed with 50μl of cell suspension, and 10 μL of this loaded into a hemocytometer for cell counting. The cell suspension was plated in Astrocyte Medium (1xN2 Supplement, 2 mM Glutamax, Penicillin/Streptomycin, supplemented with 5ng/ml EGF). Cells were fed this media every other day until differentiation as determined by confocal microscopy in a subset of cultures using GFAP staining to identify astrocytes. Hereafter, differentiation media (Astrocyte Media plus 1 mM dbcAMP) was used twice weekly.

### *In vitro* PDT

Tumor cell lines, primary cortical neurons and primary astrocytes were plated on black-walled 96 well plates at densities of 15,000, 50,000, or 25000 cells depending on the cell line and allowed to grow for 2 or 12 days prior to PDT. On the day of PDT, cells were incubated with ALA (Sigma-Aldrich, St. Louis, MO, USA) at concentrations from 0–6000 μM with each plate containing a solvent (ddH_2_0) and a cell death control (4% Methanol). Cells were incubated with ALA for 4 hours followed by rinsing to remove unbound ALA and PpIX prior to light exposure.

For Normothermia tissue cultures were maintained in an incubator maintained at 37°C whereas hypothermia was achieved by setting an incubator to 32°C while keeping CO_2_, relative humidity, and oxygen at standard values of 5%, ~95%, ambient respectively. For hypothermia, the tissue cultures were placed 2 hrs before, and 2 hrs post light exposure into the incubator set to 32°C. Measurements showed that plates reached 32°C before light irradiation and maintained the target temperature ± 1°C for 5 min at room temperature during light illumination. Hypothermic tissue cultures were returned to the incubators set at 32°C for another 2 hours. At this time the hypothermia tissue samples were returned to a normothermia incubator.

PDT was executed using a custom-built lightbox containing one LED emitting 635nm (Newark Corp, Palatine, IL, USA) per well providing an irradiance of 75 mWcm^-2^, requiring 170 sec for 12.75 Jcm^-2^ radiant exposure. Cell viability was measured 24 hours later using the Presto Blue metabolic assay (Invitrogen Corp., Carlsbad, CA, USA)[27], employing a Flexstation 3 plate reader (Molecular Devices, Sunnyvale, CA, USA) at ten reads per well.

The survival percentage, normalized at 100% survival (ddH_2_O) and 0% survival (4% methanol), was plotted versus ALA concentrations on a logarithmic scale and a non-linear, sigmoidal, regression analysis was performed, using GraphPad Prism Software (Version 6.0 Mac, GraphPad, La Jolla, CA, USA) determining the LD_50_,. The tested null hypothesis was that the LD_50_ slopes of the hypothermia and normothermia curves are not significantly different.

### *In vivo* study design and humane endpoints

In total, 34 animals were used for the *in vivo* experiments. Animals were followed for up to a period of 30 days including both tumor generation until PDT (approximately 10 days) and survival following PDT (up to 17 days). The thirty days also includes rats used for the T_2_ mapping experiment involving PDT on a healthy brain.

For all procedures that were survival, animals were pre-treated with buprenorphine (0.05 mg/kg) and dexamethasone (5 mg/kg IV–PDT only) prior to surgery (using aseptic techniques). Following surgery, animals were given buprenorphine every 8 hours (0.05 mg/kg) for 72 hours, while dexamethasone was given for 6 days post PDT at once a day 2 mg/kg. If indicated post-treatment, the 72 hour period of supportive analgesia was provided, including buprenorphine and meloxicam at 0.05 mg/kg and 1 mg/kg, respectively.

Animals were monitored twice daily for the duration of the study, and a clinical monitoring sheet was used for humane endpoint determination. Once an animal reached humane endpoint, it was euthanized immediately, brain resected and sent to histology. The monitoring sheet examined 6 parameters (activity and mentation, general appearance, posture, weight and condition, respiratory quality, and neurological signs). The scores were assigned a value between 0–3 with 0 being considered ‘normal.’ At a score of 3, supportive care was assigned (soft food, fluid support, and KMR) and a score of 9 was considered humane endpoint. Monitoring was performed by a mixture of veterinary technicians and lab staff.

All animals were euthanized by an intracardiac injection of sodium pentobarbital (>120 mg/kg) under deep anesthesia. Of the total cohort, 4 animals were found dead within the cage between the evening observation period and the morning observation period. No animals need to be euthanized or died unexpectedly following tumor induction or PDT in the study.

### RG2 tumor inoculation

RG2 tumors were generated by injection of 5000 cells in sterile DPBS via a burr hole 1 mm into the neocortex of CDF Fischer rats (Charles River Laboratories, Wilmington, MA, USA), 3 mm from the midline and 3 mm from the bregma utilizing a stereotactic frame. Following injection, animals were monitored using T_2_w and Gd-enhanced T1w MRI until the tumors reached 4 mm diameter for treatment. An overview of the in vivo timelines for the tumor bearing rat experiments in provided in S1 Fig.

### *In vivo* and *ex vivo* PpIX concentration quantification by point spectroscopy and tissue solubilization

Glioma and normal brain PpIX concentration were quantified *in vivo* using a point spectroscopy method and ex vivo by tissue solubilization. Point spectroscopy utilizes fiber optical delivery[28, 29] measuring local absolute PpIX concentration. Two 3 mm burr holes ipsi- and contralateral to the tumor were drilled to the dura and the 1 mm probe head lightly pressed against the tissue. The 405 nm excitation light is strongly absorbed, and the interrogation volume is limited to 1 mm^3^ adjacent to the probe tip.

For PpIX quantification by the tissue solubilization method, animals were euthanized, and 50–100 mg tissue samples harvested [14] and, mechanically homogenized followed by a chemical digestion prior to dilution for uniform excitation of the homogenate. Fluorescence emission after 405 nm excitation was performed using a Fluorolog spectrofluorometer (Horiba Scientific, Kyoto, Japan). The fluorescence spectrum was decomposed into PpIX and endogenous fluorophores, including reduced nicotinamide adenine dinucleotide and Flavin adenine dinucleotide, using standard emission spectra of these fluorophores. Comparing the homogenate’s PpIX fluorescence against a PpIX standard concentration (0.05 μgmL^-1^), provided the tissue ‘s PpIX concentration.

### MRI scanning and analysis

MR imaging used a 7 Tesla Biospec 70/30 USR system (Bruker Corporation, Ettlingen, DE), equipped with B-GA12 gradient coil insert, 7.2 cm inner diameter linearly-polarized cylindrical volume RF transmission coil, and a 4-coil phased array surface receiver coil for RF reception all part of the Biospec line. After anesthesia induction by 2% isoflurane (in O_2_ at 0.5 Lmin^-1^), rats lay prone, breathing via nose cone and resting on a 37°C water bed for imaging. Respiratory was monitored by pneumatic pillow (SA Instruments, Stony Brook, NY). When required, the tail vein was cannulated by a 27G catheter for injection of 90 μl gadolinium- MR contrast (Gd-DTPA, Magnevist, Bayer Corporation) using an injector pump (PHD 2000, Harvard Apparatus).

T_2_-weighted imaging used a Rapid Acquisition with Relaxation Enhancement (RARE) technique with an 85 ms echo time (TE), 5200 ms repetition time, with a RARE factor of 18, and 5 averages, requiring ~ 3min imaging time. T_1_-weighted imaging used the RARE technique with 9.6 ms TE, 1000 ms TR, a RARE factor of 2, and 4 averages requiring 4 min 16 sec.

MRI tumor volume assessment was performed on days -4, -1, 10, and weekly after that, and included multi-slice 2D T_2_-weighted and contrast-enhanced T_1_-weighted acquisitions. The geometric features of both acquisitions were matched (25.6x25.6 mm field-of-view, 128x128 matrix, 0.2x0.2 mm in-plane resolution, at least eighteen 0.5 mm thick slices collected).

To assess brain and intratumoral edema maps, quantitative T_2_ maps, were acquired on days 2, 10, and 28 using a multiple spin echo technique. At least 9 contiguous tumor containing slices were collected. The imaging parameters were: 48 echoes ranging from 12 to 576 ms; with a 12 ms refocusing interval; TR = 8000 ms; 25.6x25.6 mm field-of-view; 100x100 matrix; 0.256x0.256 mm in-plane resolution; 1 mm thickness; acquisition time 10 min 8 sec.

Independent researchers drew region of interest (ROIs) on T_2_ maps using MIPAV (version 7.2.0 CIT-NIH, Bethesda, MA, USA). Mean, and Standard Deviation of T_2_ and ROI volumes were exported into GraphPad Prism for 2-way ANOVA testing between the treatment groups. Mean baseline T_2_ values were derived from the treatment area’s contralateral side across all image slices excluding the lateral ventricles. At day 2, ROIs were drawn on the treatment volume across all image slices and transcribed to the next two imaging time points to assess the amelioration of inflammation and edema over time.

### *In vivo* PDT

PDT was performed on all rats when their tumors reached 3–4 mm diameter to test for neuroprotective effects of hypothermia, including 8 non-tumor bearing rats and 18 RG2-inoculated rats. An IP injection of ALA (pH 6.8, 62.5–125 mgkg^-1^) was given 4 hours prior to light delivery. Two drug doses were required because 125 mgkg^-1^ is an often-reported drug dose which under hypothermia has proven excessive in most animals. Two hours post-ALA, animals were anesthetized and placed on a heating pad set to either 38 or 32°C and kept there for 4 hrs. Animals were monitored continuously, and temperature measurements logged every 5 min using the Luxtron system (LumaSMART, LumaSense Technologies, Inc. Santa Clara, CA) and recorded manually using the digital rectal thermometer. Results demonstrated a 1.5–2°C difference between the temperature recorded with the rectal probe and the averaged Luxtron sensor measurement (S2 Fig). For our experiments, we utilized the rectal temperature probe plus an offset of 1.5°C to mark the correct hypothermia conditions. The temperature took approximately 1.5–2 hours to approach a mild hypothermia level (32–34°C), and for subsequent experiments, hypothermia was initiated 2.5 hours before PDT. The maximum permissible anesthesia time without intubation, 5 hrs, determined the total duration of hypothermia in these experiments, which was achieved 1 hour prior to PDT and maintained for 2 hrs post irradiation.

Animals were irradiated with 24 J of 635 nm light (an irradiation time of 22 mins 13 secs), delivered via an isotropic emitter inserted 1 mm below the dura in the superior portion of the tumor, rather than in its center. Following PDT, rats received 2 mgkg^-1^ dexamethasone daily for 5 days (derived from [30]) and were followed until they reached a determined protocol endpoint. Dexamethasone co-therapy was required because its absence resulted in extensive inflammation and treatment-related mortality in a pilot study.

### Histology samples and preparation

Post euthanasia, brains were harvested in whole and placed in 10% formalin. Brains were cut along a transverse plane 3 mm anterior and 3 mm posterior to the emitter insertion site, resulting in a 6 mm thick section containing tumor and its contralateral side. The section was mounted, embedded in paraffin from which 6 μm sections were cut, mounted onto slides, and stained with either H&E or GFAP. The latter is an astrocytic marker, which increases in intensity following astrocyte activation or astrogliosis.

Immunohistochemistry stained slides were scanned at 20x magnification in their entirety, generating a digital image, using an Aperio ScanScope XT (Leica Biosystems, Concord, ON, CA) brightfield scanner. The analysis used Aperio ImageScope software (Leica Biosystems).

### Statistical analysis

Determination of the LD_50_ concentrations *in vitro* was based on a non-linear regression analysis performed using GraphPad Prism Software (Version 6.0 Mac, GraphPad, La Jolla, CA, USA). To test the normality of the data, using p < 0.05 as a cut-off, residuals were plotted (D’Agostino and Pearson), and a Brown-Forsythe and Bartlett’s tests were performed to test the assumption of equal variance between the sample groups. The difference in the tissue accumulations of PpIX was tested using the Student’s T-test (p < 0.05 for significance). The *in vivo* Kaplan-Meyer survival curves were analysis using the Mantel-Cox Log-Rank test. To compare post-PDT inflammation from T_2_ maps, ROIs were drawn by multiple researchers using MIPAV (version 7.2.0 CIT-NIH, Bethesda, MA, USA) and propagated across time-points. The information obtained includes average and standard deviation T_2_, the number of voxels, and total volume which was exported to GraphPad Prism to compare ROI regions between therapeutic groups. Testing of T_2_ values was performed using One-way ANOVA with Turkey correction for multiple comparisons. ANOVA’s were performed after testing data for normality followed by a Brown-Forsythe and Bartlett’s tests to determine for equal variance between treatment groups. If an unequal variance was found, the Welch Test was performed to test for statistical significance.

## Results

**Fig 1:**
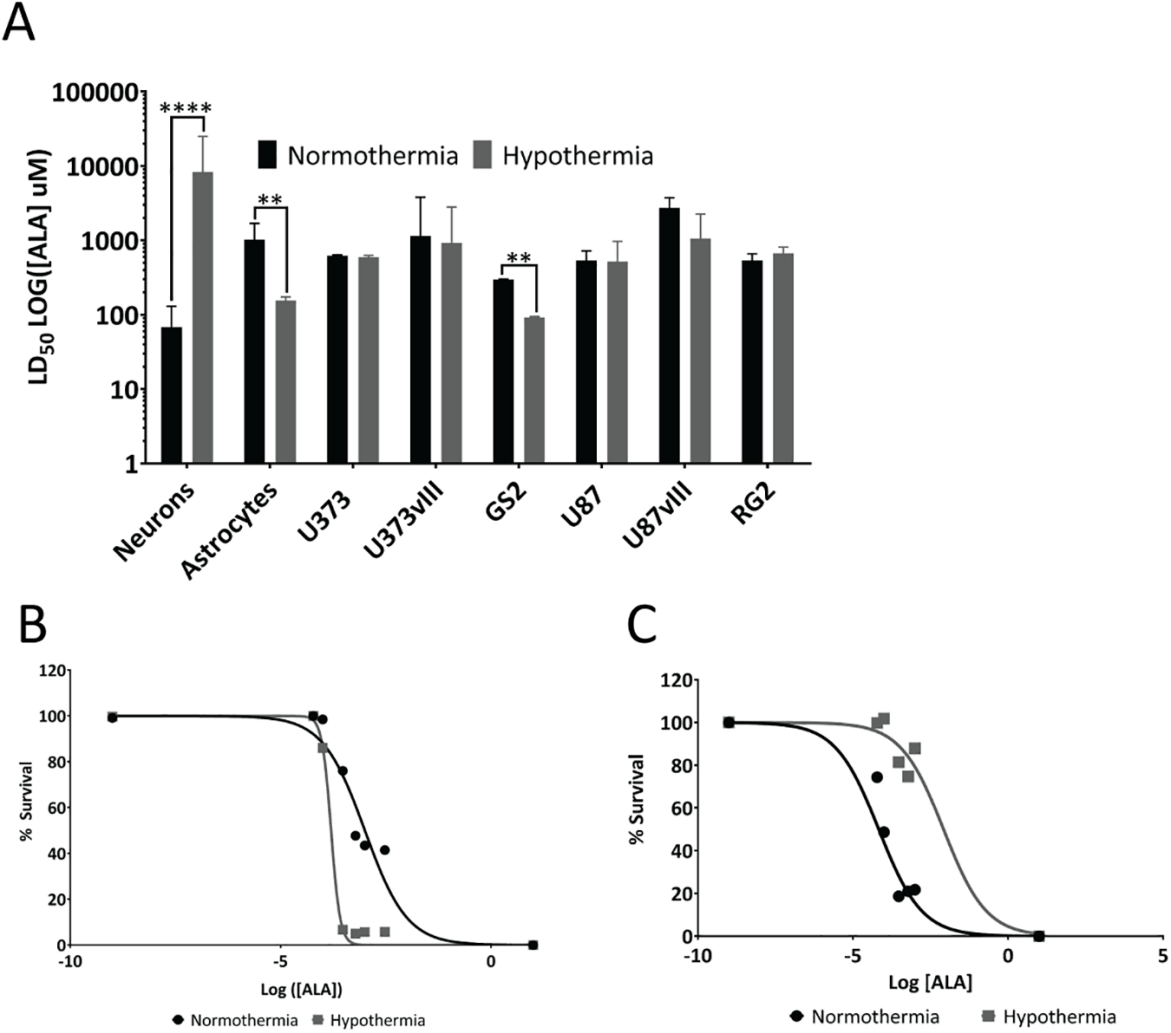
Mild hypothermia modulates PDT responsivity of cell lines and primary neuronal cells *in vitro*. A) LD_50_ values of 5 glioma cell lines, one purported glioma cancer stem cell line (GS2), as well as primary murine neurons (DIV 13) and astrocytes of PDT with and without hypothermia, note LD_50_ of ALA, was chosen as a surrogate for PDT dose in these studies. Hypothermia altered LD_50_ significantly from primary neurons and astrocytes, as well as GS2 cells (p<0.05, n = 3). B) Normalized dose-response curve of primary astrocytes and C) of primary neurons.

Survival assessment based on the Presto blue metabolic assay demonstrated that cultured primary rat neurons experienced significantly sparing of PDT-induced cell death when using ALA concentration [μM] LD_50_ as the PDT dose surrogate for constant radiant exposure. The normothermia LD_50_ was 68 μM whereas the hypothermia LD_50_ was 8000 μM or 2 orders of magnitude higher (p<0.05, Fig 1A).

Interestingly, primary astrocyte survival was significantly lower following hypothermia PDT (LD_50_ = 15 5μM ALA) compared to normothermia PDT controls (LD_50_ = 1000 μM ALA) (p<0.05, Fig 1A and 1B). While the dose-response curve for primary neurons retains its shape it is shifted to higher ALA concentrations, Fig 1C; primary astrocytes treated under hypothermic conditions respond with a much sharper dose-dependence to PpIX mediated PDT. This may be an indication that these cells’ ability to cope with cytotoxic stress was altered. There was no significant difference in the LD_50_ of any glioma cell lines, except GS2 stem cells, presenting a normothermia LD_50_ = 298 μM ALA vs. LD_50_ = 91 μM for hypothermia (p<0.05, Fig 1A).

Initial *in vivo* experiments using an ALA dose of 125 mgkg^-1^ resulted in a very strong PDT response in the hypothermia group resulting in the animals’ death. Hence, all *in vivo* experiments except the uptake studies are based on an administered ALA dose of 62.5 mgkg^-1^.

**Fig 2:**
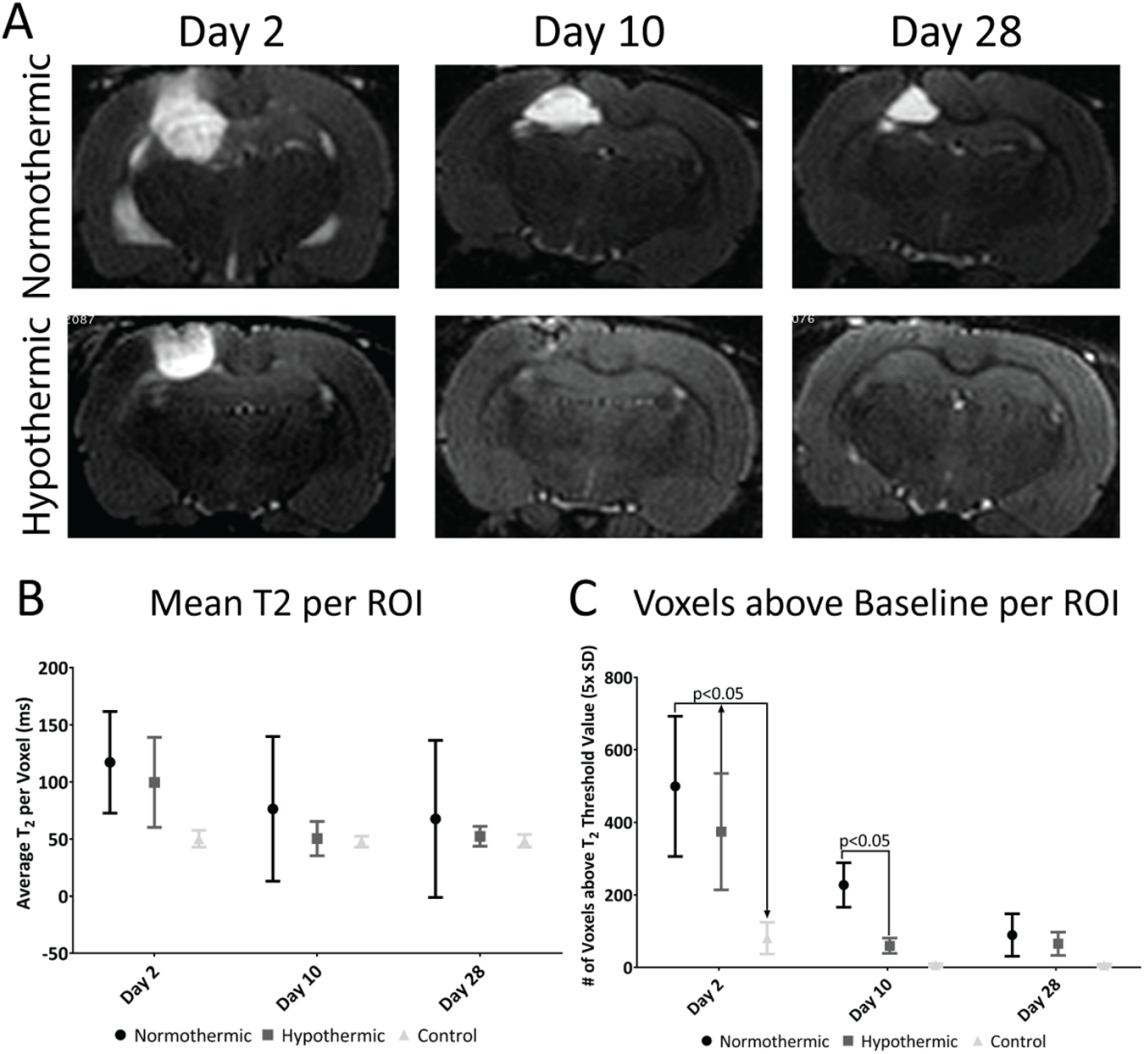
Hypothermia reduces volume and intensity of edema/inflammation on the healthy brain following PDT. A) T2 weighted images of central slice regions (areas with the highest amount of inflammation) for each of the treatment conditions. B) Mean T2 of the ROIs of T2 maps for each of the treatment conditions (p>0.3, n = 4 animals) C) Number of voxels above baseline in each treatment group versus the contralateral side control (no PDT). (p<0.05, n = 4 animals).

Fig 2A shows quantitative T_2_ maps in non-tumor bearing rats, at 2, 10 and 28 days post-PDT under hypothermic and normothermic conditions. At 2 days post-PDT, the acute responses presented with equivalent T_2_ elevations between groups (p>0.3, n = 4 rats per group) and similar appearances between groups. At day 10, the ameliorated reduction of inflammation/edema in the hypothermic cohort approached significance (normothermia T_2_ = 76±63 ms; hypothermia T_2_ = 50±15 ms, p = 0.06). The data was dominated by a reduction of the voxel count with prolonged T_2_ values (50 ms over 47 ms baseline) for hypothermia versus normothermia (227±61 voxels versus 60±21 voxels, p<0.05), as shown in Fig 2C. At day 28, ROI T_2_ values and the number of voxels above baseline were no longer significantly different between the group, (normothermia: T_2_ = 67±63 ms; hypothermia: T_2_ = 52±8 ms; p>0.9 for both parameters). T_2_ value elevation above baseline was not observed in control animals which did not undergo PDT treatment (Fig 2B).

**Fig 3:**
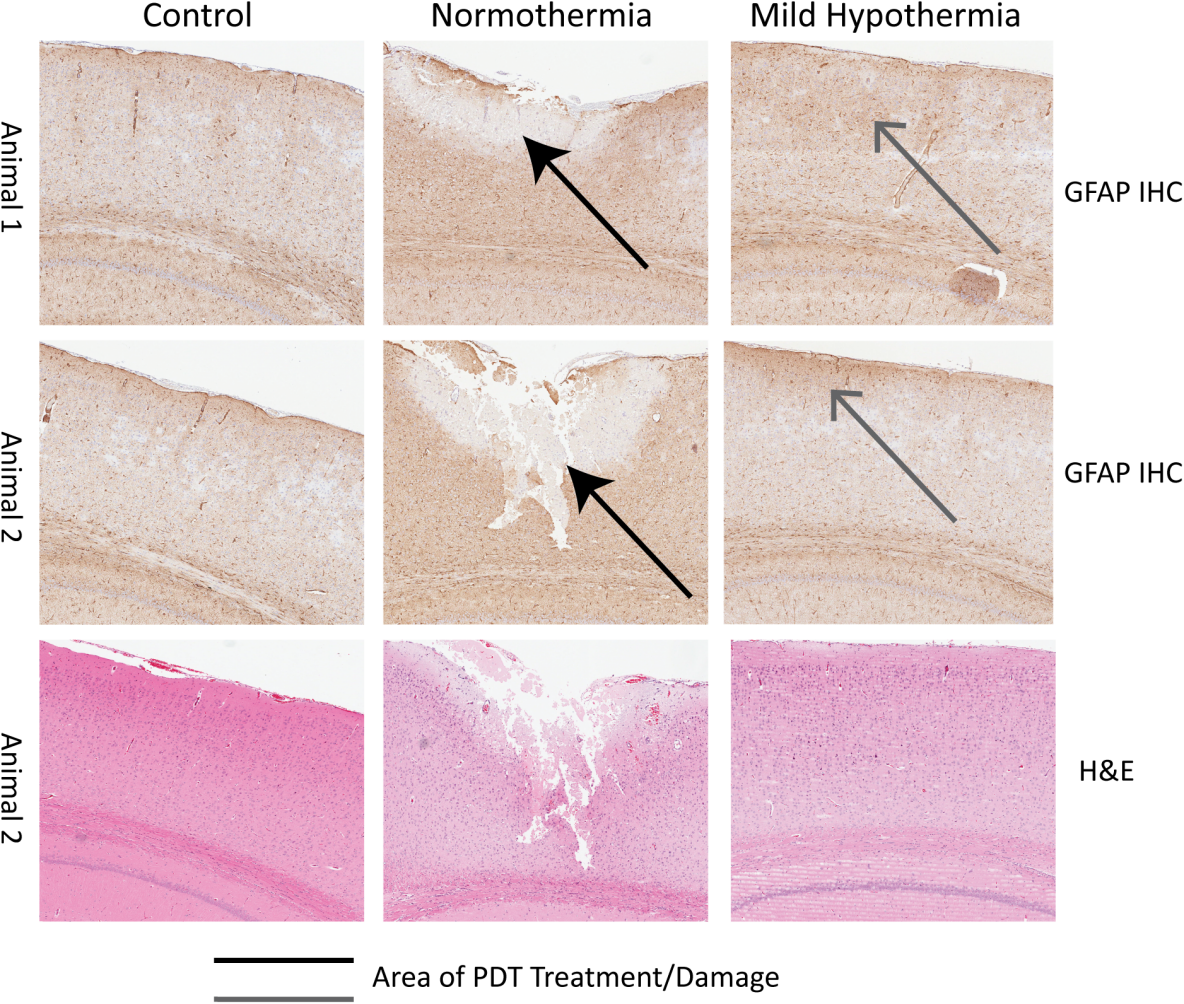
Hypothermia reduces lesion volume on the healthy brain following PDT treatment. A) Extracted regions of GFAP staining used in the analysis. For each subgroup, 2 animals were subjected to PDT and followed for 10 days using MRI. Note: Normothermia animals demonstrated some necrosis that was not seen in the other subgroups (The top and bottom panels represent different animals) (blue arrows mark out increased GFAP staining, red arrows mark out cell death).

Histological sections of PDT-treated cortical brain regions, obtained at day 10 post-PDT and subject to reactive astrocytes IHC-GFAP staining, are shown in Fig 3A. GFAP staining of PDT-treated regions indicated that following hypothermia brains presented with astrocyte invasion into the PDT-treated area, suggestive of reactive gliosis and a glial limitans being established in cortical layer 1 with astrocytes also found in layers 2 and 3 (open arrow). This was not observable under normothermia PDT. Additionally, for hypothermia PDT a profound tissue sparing was noted compared to normothermia PDT, indicated by the closed arrows in Fig 3. The damaged area in each section was summed for each of n = 3 animals respectively, resulting in 3.9*10^−6^±1.1*10^−6^ m^2^ for hypothermia and 6.3*10^−8^±2.5*10^−8^ m^2^ for normothermia PDT (n = 3, p< 0.01).

**Fig 4:**
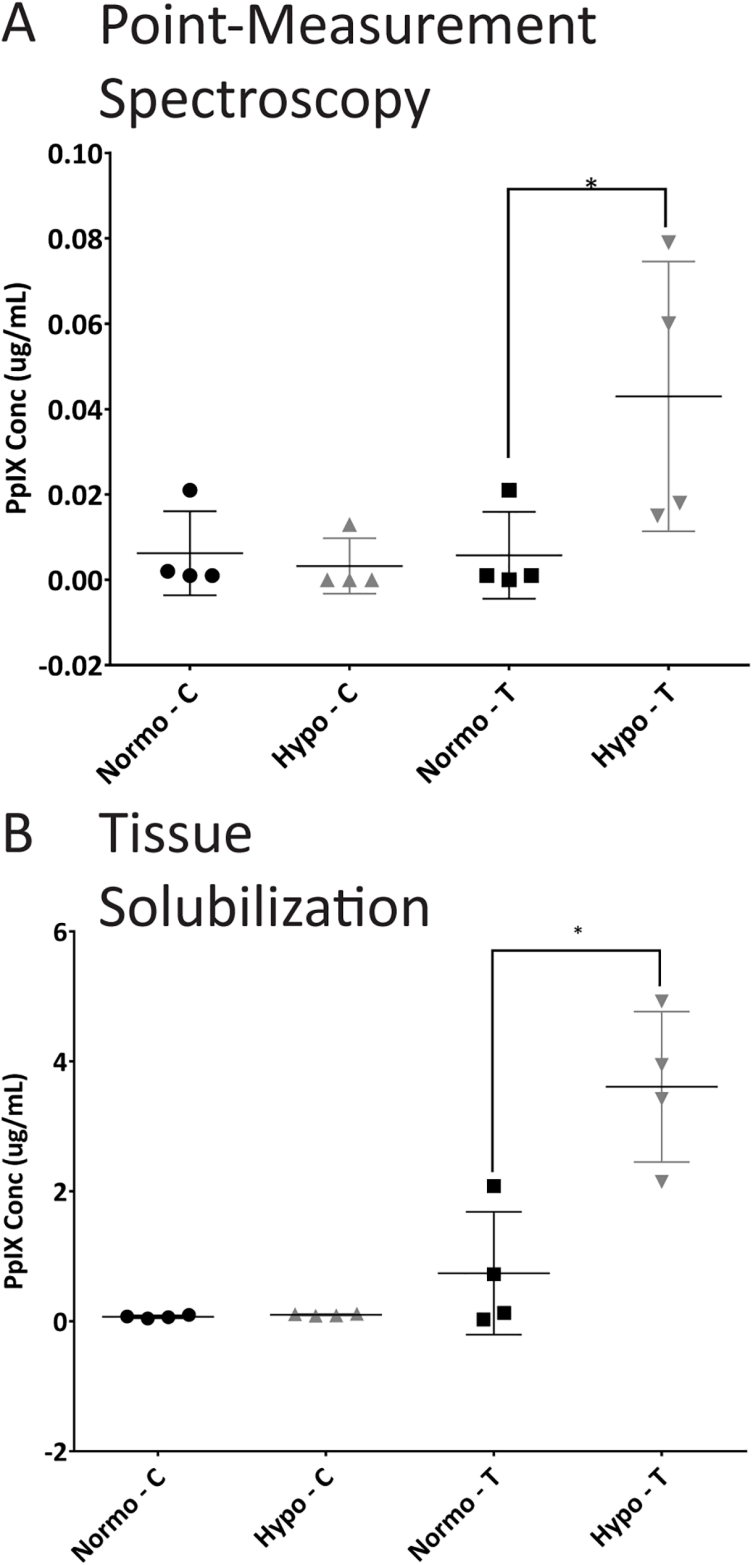
PpIX fluorescence increased in tumor cells following mild hypothermia treatment. A) Point spectroscopy of PpIX fluorescence in live animals. Capital C stands for contralateral (non-tumor bearing hemisphere), while capital T is for the tumor-bearing hemisphere (p<0.05, n = 4). B) Tissue solubilization data of PpIX fluorescence between the same animals from panel A, following euthanasia (p<0.05, n = 4 animals).

Following the initial non-tumor bearing rat studies, tumor studies commenced utilizing the RG2 glioma model, whereby hypothermia resulted in increased PpIX concentration at four hours following ALA administration, compared to normothermic controls. Fiber based point spectroscopy (Fig 4A) showed four-fold higher PpIX accumulation in hypothermia exposed tumors compared to normothermic tumors (0.04±0.02 μgmL^-1^ versus 0.01±0.005 μgmL^-1^, p<0.05, n = 4). Sub-detection levels of PpIX fluorescence were registered in the contralateral brains. Tissue solubilization (Fig 4B) revealed a similar 5-fold PpIX concentration increase in hypothermia versus normothermia exposed tumors at the same time-point, reporting 3.6±0.6 μgmL^-1^ and 0.74±0.5 μgmL^-1^, respectively (p<0.05 for n = 4) with sub-detection levels of PpIX in the contralateral sides. The differences in absolute concentrations between the techniques were caused by the ~ 1 μL sample volume of the optical probe, undersampling the tumor volume.

**Fig 5:**
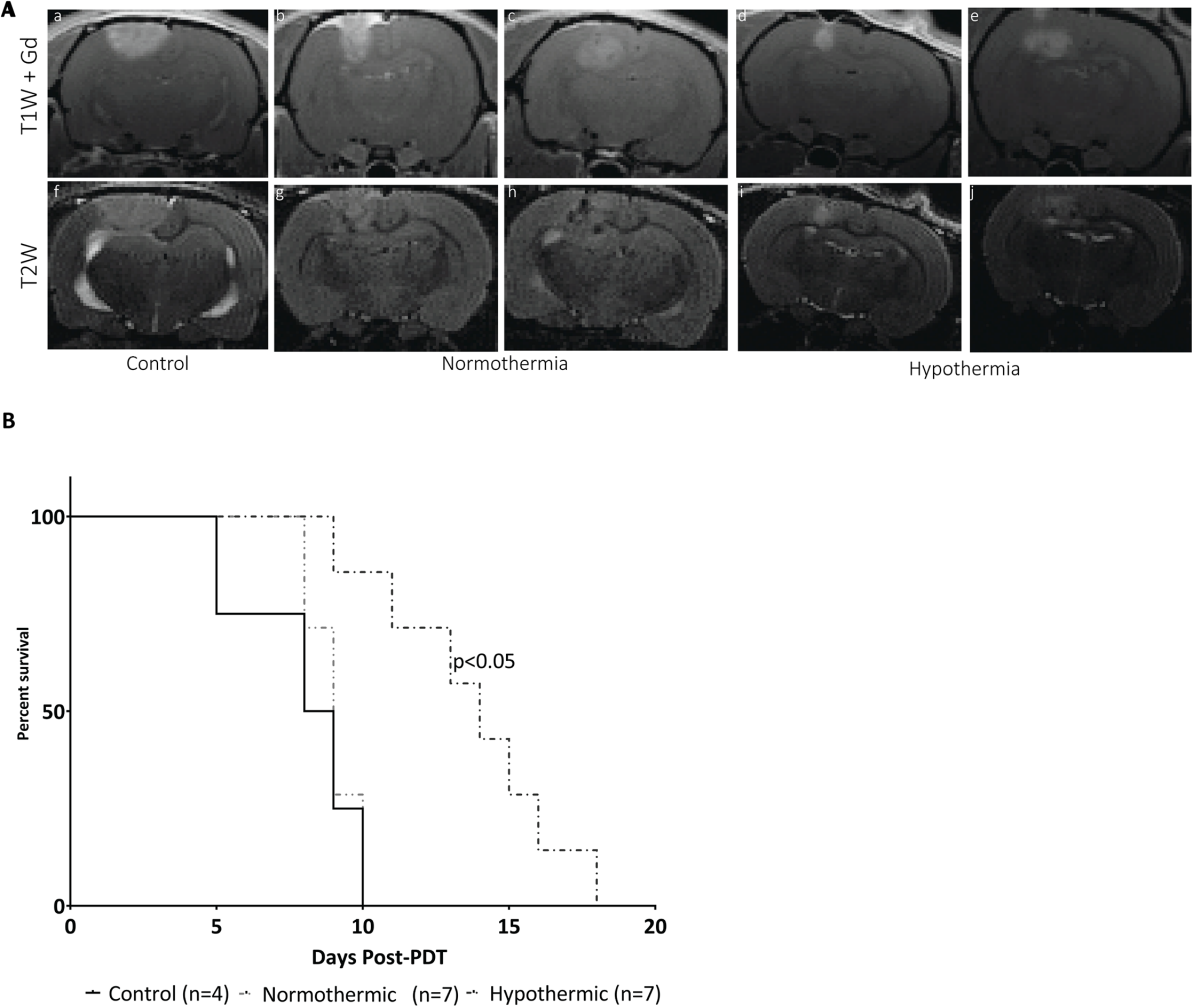
Mild hypothermia leads to significant increases in survival following PDT of RG2 tumors. A) MR images of central slices of 5 different RG2 tumors before PDT treatment (images were taken at day -1) including (a-e) contrast-enhanced T1w; and (f-j) T2w images. B) Survival post-treatment of animals in each cohort (solid black line–control, short broken line–normothermia, broken line–hypothermia).

Untreated RG2 tumors measured 3–4 mm diameter at day 8–10 post tumor induction (Fig 5A) and reached a predetermined endpoint at day 15–17 approximately 8.5 days later, which represents the control median survival time for comparison with the PDT treated groups. Experiments demonstrated a significant survival increase, p <0.05, according to the Mantel-Cox Log-Rank test, for RG2 bearing rats when treated with hypothermia PDT 14 days versus 9 days for the normothermia PDT and 8.5 days for the untreated controls (Fig 5B).

**Fig 6:**
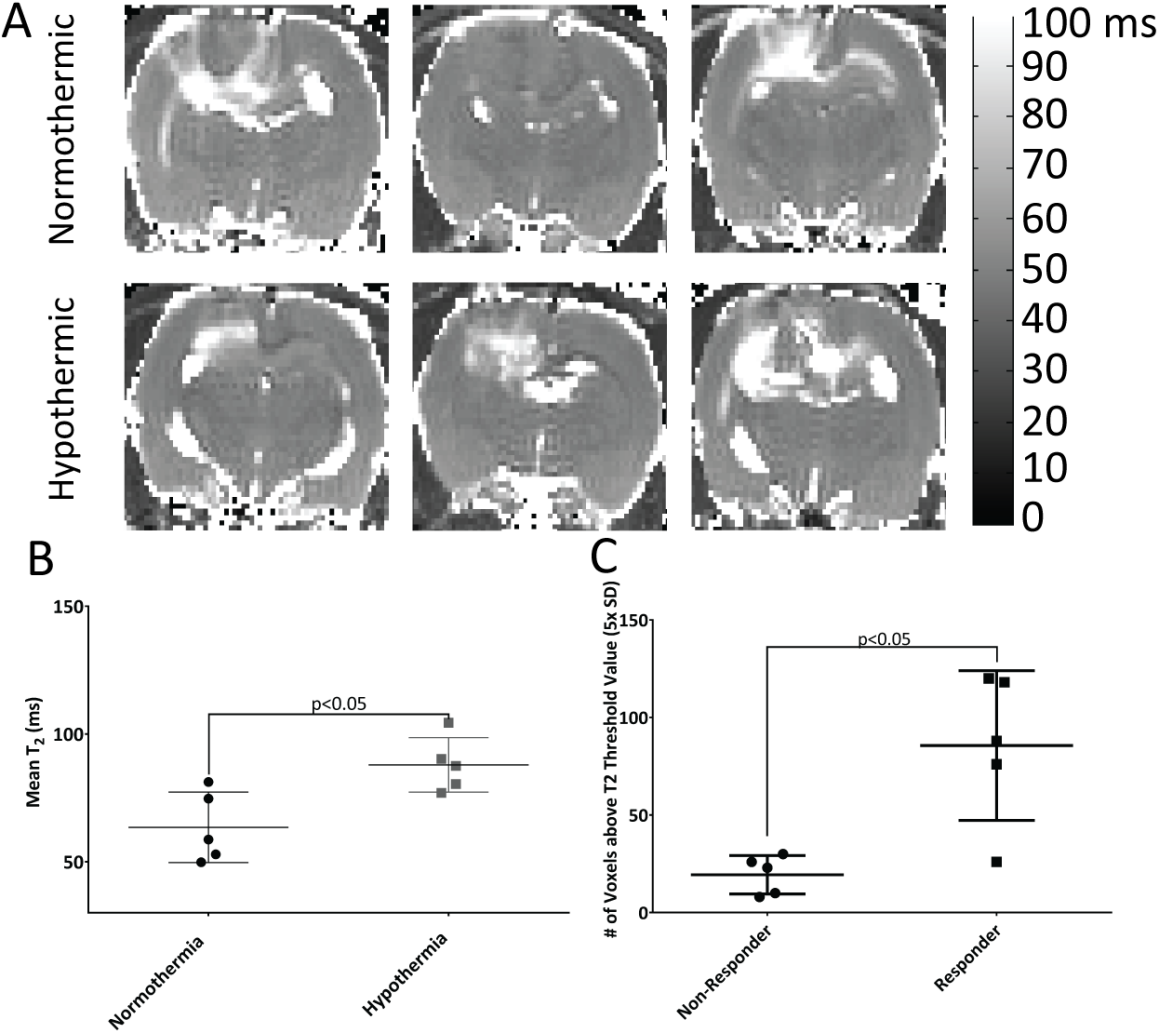
Mild hypothermia leads to increases in edema/inflammation through the tumor volume following PDT treatment. A)T2 maps of 3 animals for hypothermia and normothermia PDT-treated at day 3. B) Mean T2 of the ROIs of T2 maps for each of the treatment conditions around the tumor treated area (p<0.01, n = 7 animals per group). C) The volume of inflammation reported as some voxels above baseline T2 in the PDT-treated area (p<0.05, n = 7 animals per group).

MRI T_2_ mapping of the tumor at day 3 post-PDT suggests greater edema/inflammation in tumors treated by hypothermia PDT compared to normothermia-PDT rats (Fig 6B) with hypothermia PDT mean T_2_ values significantly longer (p<0.01, n = 4) then post normothermia. Within hypothermia PDT-treated volumes an average of 86±17 voxels exceeded the pre-PDT baseline+5 standard deviation T_2_ threshold whereas only 19±4 voxels did so for the normothermia, PDT-treated animals (p<0.05, Fig 6C).

## Discussion

Factors which modify PpIX accumulation may lead to improved FGR and PDT efficacy. This study builds on a prior *in vitro* study demonstrating hypothermia (32–34°C) leading to higher PpIX concentrations in tumor cell lines, thus allowing for higher selectivity in FGR and a greater therapeutic index for PDT[20]. For glioma invading the normal brain, PDT selectivity cannot be provided by the fluence rate (ϕ), nor the oxygen gradient, as neither will vary across the size of the micro invasions. Thus, selectivity is provided only by the difference in PpIX accumulation in the tumor versus the normal host brain and the tissue’s intrinsic responsivity to the cytotoxic dose from PDT. The tissue responsivity is given by its PDT threshold value, T. When the PDT dose, given by the light dose, or fluence rate *ϕ* [mWcm^-2^] and the photosensitizer concentration [PpIX], exceeds a threshold value T, tissue destruction occurs. To achieve selective GBM destruction up to the clinically required depth, d, the following conditions need to be satisfied.

$$\frac{T_{Tumor}\;P(0)}{{\left[PpIX\right]}_{Tumor}\;\phi (d)}<\frac{T_{Brain}}{{\left[PpIX\right]}_{Brain}}$$ (1)

whereby, the light fluence rate is a function of depth, d, provided by

$$\phi (d)=\frac{P(0)}{4\pi \mu_{s}^{\prime }d}e^{-\mu_{eff}d}$$ (2)

The PpIX concentrations are given by [PpIX]_tumor_ and [PpIX]_brain_ respectively, and the T-values are presenting the tissue’s PDT responsivity in units of photons absorbed by PpIX per unit volume. The fluence rate is given by *P(0)* the optical power delivered by the light source and its depth dependency is modulated by the tissue optical properties μ_*eff*_[cm^-1^], the effective attenuation coefficient and μ_*s*_^*’*^[cm^-1^] the reduced scattering coefficient. To maximize the PDT selectivity and hence GBM resection d in (1), must be maximized.

Hence, improving the PDT selectivity as a function of distance from the light source requires one or a combination of the following four conditions to change. First increasing the PpIX concentration in the tumor, second reducing it in the healthy brain, third increasing normal brain resilience against PDT or fourth decreasing it in the GBMs. Any significant changes in any of these four parameters would result in a beneficial change towards tumor destruction.

### *In vitro* experiments

Based on previous published quantitative PpIX imaging in live tissue cultures *in vitro* [20], we expected a higher cytotoxic cell kill as a consequence of the higher cellular PpIX concentration. However, *in vitro*, this higher cytotoxicity was not observed post-PDT, save for GS2 cells, with a reduction in LD_50_ following the largest mitochondrial PpIX fluorescence increase under hypothermia. While RG2 cells showed a similar PpIX associated fluorescence increase, it did not translate into increased hypothermia PDT responsivity. However, the observed PpIX fluorescence increase may be an artifact as RG2 cells grew in clusters and the observed fluorescence may be associated with PpIX at less sensitive cellular structures within the cytoplasm.

PDT protection provided by hypothermia to neurons, as reflected by the dramatic increase in the LD_50_, even exceeding the most PDT resistant cell lines with constitutively active EGFR signaling (U87vIII and U373vIII) [31] is exciting. Hence, an increase in the distance (d), over which the PDT dose and selective can be provided, according to Eq. 1 can be driven by an increase in T_Brain_, and an increase in [PpIX]_tumor_. Conversely, the significant decrease in hypothermia PDT LD_50_ in normal astrocytes is of concern. Reactive astrogliosis following neuronal insults could be beneficial or a hindrance to long-term neuronal survival, as reactive gliosis can both contain or lead to neuronal tissue cell death in an area larger than initially injured[32].

### *In vivo* experiments

While the timing of hypothermia induction and cessation relative to light exposure was similar for *in vitro* and *in vivo* studies, the actual time-course of hypothermia was different due to the thermal capacity, and hemodynamics of an animal subject, which may have modified the response of astrocytes and normal neural tissue.

The increased PpIX fluorescence from RG2 tumors *in vivo* following hypothermia is expected from our *in vitro* studies but contrary to studies on skin demonstrating an increase PpIX accumulation at 38.5°C versus 29°C [33]. This may be attributable to the much lower temperature in that study; 29°C being considered outside the range of mild hypothermia and not examined in this work. The higher PpIX accumulation can improve contrast to detect micro-invasion and may allow tumors with a weak accumulation of PpIX, such as Grade II or III gliomas to be detected easier, thus improving patient outcome [34–36].

PpIX accumulation is governed by a multitude of effects, ranging from tissue stiffness[37], enzymatic activity, transmembrane transport of ALA and PpIX and hypothermia effects on BBB permeability. Moan et al.,[38] demonstrated increased PpIX fluorescence from ALA exposed skin at 37°C compared to 31°C, citing diffusion of ALA through the skin and the activity of porphobilinogen deaminase one of the key enzymes in PpIX synthesis, so both are not temperature dependent. Qualitative assessment of mild hypothermia on BBB permeability showed no effects in either isoflurane- or pentobarbital-anesthetized rats [39]. Indeed, BBB permeability appeared to be reduced during hypothermia for several means of opening the BBB such as Oleic acid infusion[40], traumatic brain injury[41] or hyperosmolar solutions. Hypothermia should not affect ALA diffusion across the BBB significantly [42, 43]. However, PpIX does not cross the BBB by diffusion and as it is quickly excreted from glioma cells[44], tightening of the BBB could cause added retention of PpIX in the healthy brain.

Prolonged T2 relaxation times (MRI T2 values) in images from tumor-free animals acquired at days 2, 10, 28 post PDT, demonstrated the presence of free water protons not associated with structural proteins, which is characteristic of edema and inflammation. An inverse correlation between PDT-induced inflammation and animal survival was noted, similar to benefits from steroids (prednisone) ameliorating inflammation resulting in longer survival[30]. To mitigate excessive, immediate, post-therapy inflammation, animals were given dexamethasone for 5 days starting at PDT, and no mortalities or signs of neurological distress were noted during that 28 days period. Steroids co-therapy reflects the current standard of care following brain tumor diagnosis[45] but is in contrast to early PDT trials which commonly incorporated a corticosteroid holidays during treatment[46].

GFAP and H&E staining at day 10 following PDT demonstrated substantial differences in both astrocyte recruitment and cell death between hypothermia and normothermia PDT groups. The lack of apparent tissue death in the hypothermia group is promising, and although there is GFAP invasion into the PDT damaged area, the tissue is spared, enabling subsequent therapeutic strategies towards improving overall brain function. The micrographs suggest the presence of astrogliosis and glial limitans. However, its presentation is not as prominent as in stroke. Regarding improved PDT efficacy and FGR selectivity, hypothermia tissue sparing suggests an impetus towards evaluating PDT doses exceeding those used here in anticipation of clinical improvement in survival for higher radiant exposures [46, 47].

The *in vivo* and *in vitro* results suggest a strong neuroprotective effect for neural tissues, including neurons, by hypothermia. The mechanism may be similar to those from stroke like-models, whereby the volumes of inflammation and damage were reduced by half[48, 49], albeit some studies did not employ corticosteroids. The data confirm and extend work by Dereski et al.[21] who showed limited neuronal damage for Photofrin mediated hypothermia PDT, suggesting that the hypothermia effect is photosensitizer independent.

The survival increase in tumor-bearing animals following hypothermia PDT (Fig 5) is encouraging, particularly because it is readily translatable into the clinic. The close to 2-fold survival time increase in this RG2 model is comparable to multiple immunology studies reporting a survival increase of 40–70% versus saline controls[50, 51]. However, these studies commenced treatment 3–5 days post-implantation, compared to the 10–12 days chosen here[50–52]. Hypothermia PDT exceeded the survival advantage of multiple anti-angiogenic therapies tested in the RG2 model[53, 54]. The general agreement between T_2_ values and overall survival of the animals is similar to that seen in other PDT studies[55, 56].

In both temperature conditions, MRI did not show complete tumor removal by T_1_w+Gd contrast and regrowth began around day 8–14 including in hypothermia animals. However, this treatment protocol is not optimized, and as animals, post-hypothermia PDT reacted well, a further increase in the PDT dose via photosensitizer or radiant exposure is feasible. An attempt to increase the dose by a factor of 4, piloted in 4 rats, led to the animals’ deaths within 12 hours of the treatment, starting with seizures immediately following PDT or from paralysis the next day. An initial speculation is that hypothermia disrupts GABAergic signaling within the brain, as suggested previously[57, 58]. That disruption, combined with an excitatory response from PDT effects on astrocytes and other cells surrounding the glioma tissue could lead to rebound hyperexcitability culminating in seizures. If that assumption is correct, administration of anticonvulsive drugs before PDT should alleviate many of the post-PDT[58] seizures and allow for higher PDT dose under hypothermia targeting larger tumors and distant micro invasions.

## Conclusions

Multiple clinically translatable benefits were demonstrated by hypothermic PpIX mediated PDT and FGR in neuronal tissue. Relevant tumor cell lines were employed *in vitro* and the RG2 model *in vivo*. For FGR applications, a five-fold increase in PpIX concentration was measured *in vivo* for the tumor cells. This contrast improvement allows for greater resection rates as smaller tumor cell clusters will become positive. For PDT, hypothermia provides two improvements. First, protection of healthy tissue may allow for potentially higher clinically light doses such that therapy may become curative for low-grade gliomas, and second that a higher PDT dose can be delivered to malignant cells due to improved synthesis or retention of PpIX. Improvements in PpIX mediated PDT selectivity for GBM therapy is based on higher PpIX accumulation in the malignant tissue and improved resistance or higher PDT threshold for normal neuronal tissues. The latter effect can also apply to other photosensitizers used in the brain as hypothermia itself demonstrated a neuroprotective benefit under acute neuronal damage settings similar to stroke patients. The prevention of tissue death at 10 days post-PDT is exciting, suggesting that hypothermia on its own could provide benefits to many therapies beyond PDT through a reduction in inflammation and subsequent neuronal cell death.

## Supporting information

S1 Fig Temporal sequence of *in vivo* experiments. Overview of *in vivo* experiments beginning at tumour injection through to humane endpoints.
(TIF)

S2 Fig Temperature measurements vs. time. Using the Luxtron FOT kit for cortex and heating pad measurements while the rectal temperature was recorded manually using a digital rectal thermometer for small animals. Temperature measurements were recorded every 5 minutes over a period of 150 minutes (n = 2).
(TIF)

S3 Fig Humane endpoint checklist. Completed humane endpoints checklist in regard to all *in vivo* studies as they pertain to this article.
(DOCX)
