# Supplementary figures and images for: ALA-PpIX mediated photodynamic therapy of malignant gliomas augmented by hypothermia
Source: PLoS One. 2017 Jul 31;12(7):e0181654. doi: 10.1371/journal.pone.0181654 (PMC5536352; doi:10.1371/journal.pone.0181654)

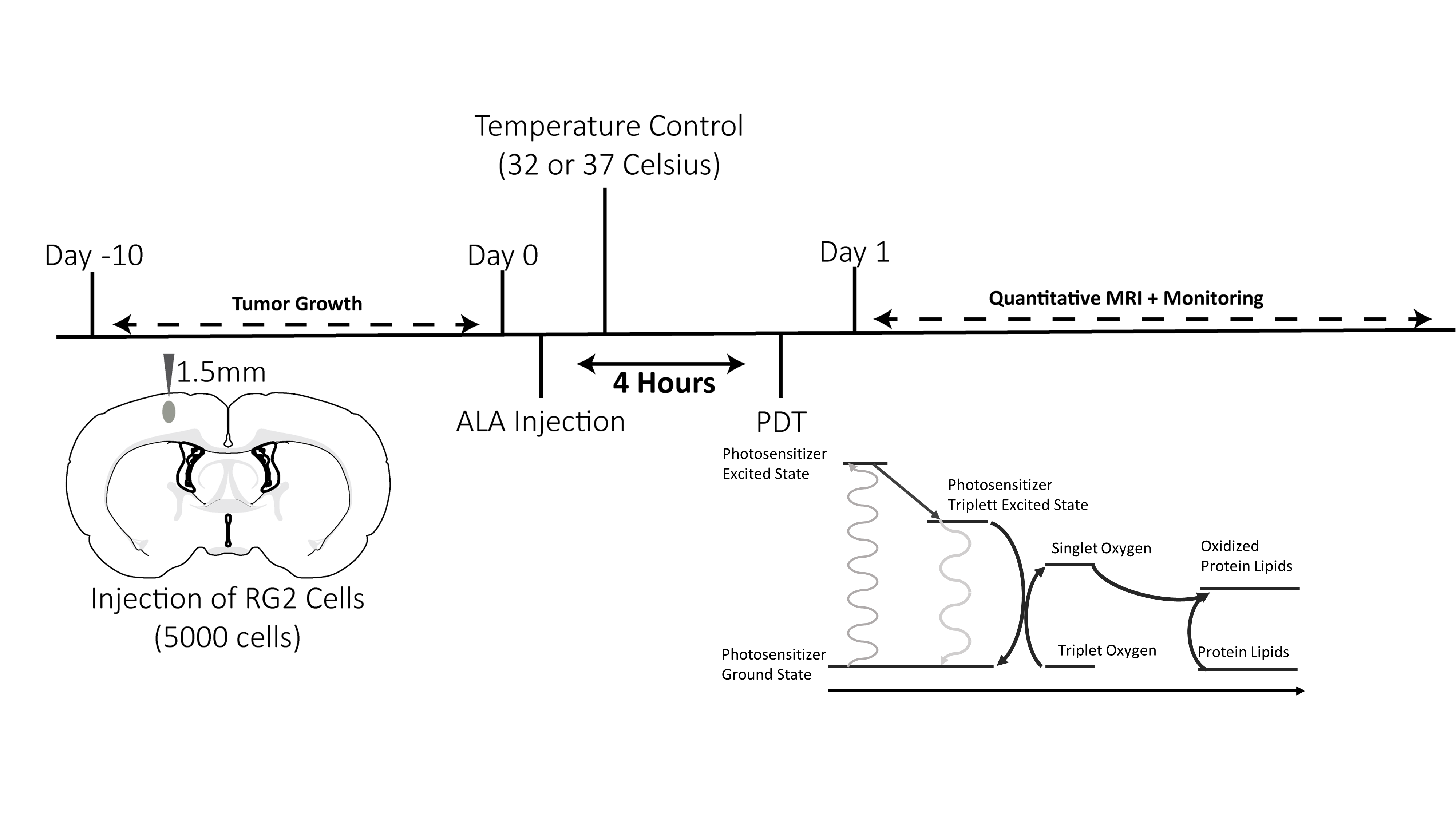

Supplement: S1 Fig — Overview of in vivo experiments beginning at tumour injection through to humane endpoints. (TIF) [file pone.0181654.s001.TIF]

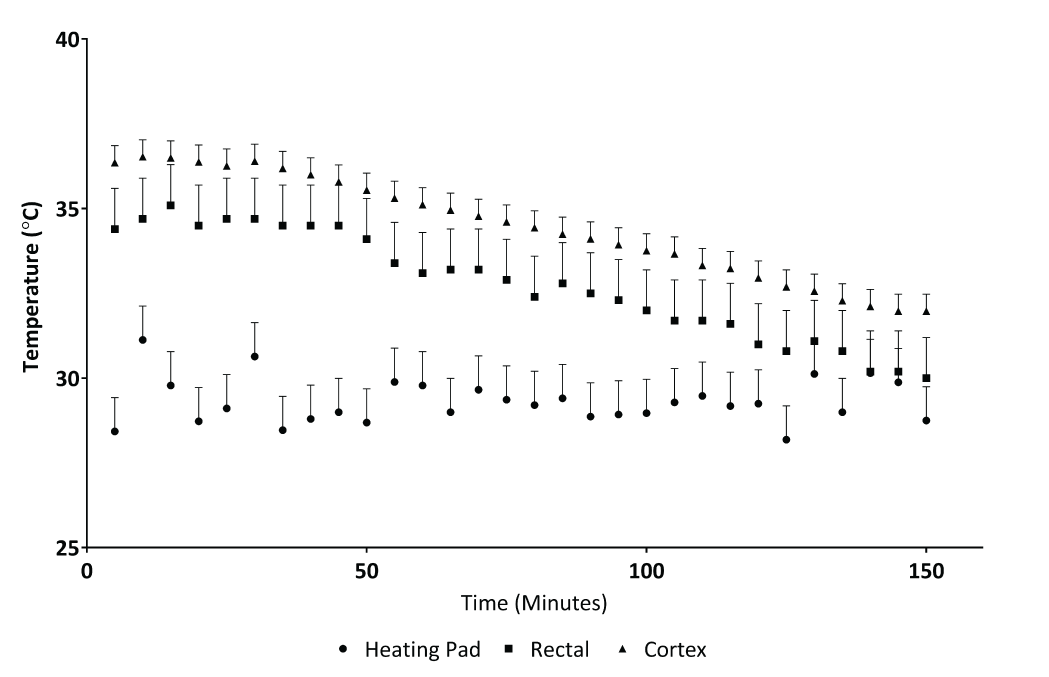

Supplement: S2 Fig — Using the Luxtron FOT kit for cortex and heating pad measurements while the rectal temperature was recorded manually using a digital rectal thermometer for small animals. Temperature measurements were recorded every 5 minutes over a period of 150 minutes (n = 2). (TIF) [file pone.0181654.s002.tif]
